# Ischemic Preconditioning in the Animal Kidney, a Systematic Review and Meta-Analysis

**DOI:** 10.1371/journal.pone.0032296

**Published:** 2012-02-28

**Authors:** Kimberley E. Wever, Theo P. Menting, Maroeska Rovers, J. Adam van der Vliet, Gerard A. Rongen, Rosalinde Masereeuw, Merel Ritskes-Hoitinga, Carlijn R. Hooijmans, Michiel Warlé

**Affiliations:** 1 Department of Pharmacology and Toxicology, Radboud University Nijmegen Medical Centre, Nijmegen, The Netherlands; 2 Department of Surgery, Radboud University Nijmegen Medical Centre, Nijmegen, The Netherlands; 3 Department of Operating Rooms and Epidemiology, Biostatistics and HTA, Radboud University Nijmegen Medical Centre, Nijmegen, The Netherlands; 4 Central Animal Laboratory and 3R Research Centre, Radboud University Nijmegen Medical Centre, Nijmegen, The Netherlands; 5 General Internal Medicine, Radboud University Nijmegen Medical Centre, Nijmegen, The Netherlands; University of Colorado Denver, United States of America

## Abstract

Ischemic preconditioning (IPC) is a potent renoprotective strategy which has not yet been translated successfully into clinical practice, in spite of promising results in animal studies. We performed a unique systematic review and meta-analysis of animal studies to identify factors modifying IPC efficacy in renal ischemia/reperfusion injury (IRI), in order to enhance the design of future (clinical) studies. An electronic literature search for animal studies on IPC in renal IRI yielded fifty-eight studies which met our inclusion criteria. We extracted data for serum creatinine, blood urea nitrogen and histological renal damage, as well as study quality indicators. Meta-analysis showed that IPC reduces serum creatinine (SMD 1.54 [95%CI 1.16, 1.93]), blood urea nitrogen (SMD 1.42 [95% CI 0.97, 1.87]) and histological renal damage (SMD 1.12 [95% CI 0.89, 1.35]) after IRI as compared to controls. Factors influencing IPC efficacy were the window of protection (<24 h = early *vs.* ≥24 h = late) and animal species (rat *vs.* mouse). No difference in efficacy between local and remote IPC was observed. In conclusion, our findings show that IPC effectively reduces renal damage after IRI, with higher efficacy in the late window of protection. However, there is a large gap in study data concerning the optimal window of protection, and IPC efficacy may differ per animal species. Moreover, current clinical trials on RIPC may not be optimally designed, and our findings identify a need for further standardization of animal experiments.

## Introduction

Ischemic preconditioning (IPC) is a potent protective strategy in which application of a brief episode of ischemia and reperfusion (I/R) results in tolerance to subsequent ischemia/reperfusion injury (IRI) [1]–[3]. The conditioning stimulus has been shown to be effective when applied either to the target organ itself (local IPC ; LIPC [4]) or to a remote organ or tissue (remote IPC; RIPC [5]). LIPC and RIPC were both originally discovered in the dog heart, and have been successfully reproduced in a variety of animal species, using various organs, *e.g.* heart, intestine, brain, liver and kidney. There is a large variety in the IPC protocols used: the preconditioning stimulus may be one continuous ischemic period, or it may be comprised of 2 or more cycles of brief ischemia. Moreover, the interval between the preconditioning stimulus and the index ischemia may vary, and positive results in animals have been found for both short intervals of a few minutes or hours (the so-called early window of protection), as well as for long intervals of days or even weeks (late window of protection).

Thus, IPC poses a promising alternative to existing treatments for IRI in humans, since current strategies to reduce this important and common clinical problem are inadequate. Next to the heart, the kidney is one of the major organs of interest for clinical application of IPC. Its high energy demand and intricate microvascular network render the kidney especially sensitive to IRI, which is a major cause of kidney injury in *e.g.* renal artery stenosis and renal surgery [6], [7]. Furthermore, renal IRI is a major cause of cardiovascular morbidity and mortality, and is associated with delayed graft function after transplantation, renal damage in cardiac and aortic surgery, and shock [8]–[11]. In animal models, both LIPC and RIPC have been shown to be effective tools to protect the kidney (*e.g.* [12], [13]).

Where do we stand in terms of the translation of IPC to beneficial treatment for patients? LIPC has not been studied in the human kidney, but several clinical studies have been conducted in the heart: a number of studies have investigated LIPC in patients undergoing coronary artery bypass grafting (CABG) surgery, which collectively show that LIPC reduces inotrope requirements, ventricular arrhythmias, and shortens intensive care unit stay [14]. For RIPC, several clinical trials have been performed for cardiac as well as renal IRI, but their outcome is not clear-cut: many studies report protective effects of RIPC after CABG surgery, heart valve surgery, or abdominal aortic aneurysm repair, but not all findings have been positive ([15]–[18] and recently reviewed in [19]).

Thus, even though the protective effect of LIPC and RIPC on renal IRI has been shown in many animal studies, translation of IPC to the clinic has, as yet, not been successful. The variety of IPC protocols used in clinical trials may be one of the reasons for this ambiguity, *i.e.* in some studies, the stimulus could have been suboptimal or incorrectly applied. There is no consensus on how many ischemic stimuli should be applied, and what the duration of the ischemic and intermediate reperfusion periods should be. It is unclear whether the early or late window of protection is most effective. Furthermore, it is unknown which patient-related factors such as age, gender or co-morbidities play a role.

Meta-analysis and systematic review of preclinical (animal) studies have previously been used to optimize experimental animal models and to improve the design of clinical trials [20]–[22]. In the case of IPC, meta-analysis on animal study data may provide valuable indicators to optimize the IPC protocol, as well as the window of protection in humans. It has been shown that proper analysis of animal experiments can also improve the decision making in whether or not to start a clinical trial. In addition, this approach can be used to perform a quality assessment of the current literature, including measures to avoid bias (*e.g.* randomization, concealment of allocation and blinded outcome assessment). As such, meta-analysis of existing literature on animal models may improve future animal research in the field, thereby contributing to the Refinement and Reduction of animal experiments, as proposed by the Animal Research: Reporting In Vivo Experiments [23] and Gold Standard Publication Checklist [24] guidelines.

This report presents innovative methods in reviewing animal studies, *i.e.* a systematic review and meta-analysis of the efficacy of IPC in experimental models of renal IRI. We set out to 1) provide a complete and systematic overview of all literature available on the effects of IPC (both local and remote) on renal IRI; 2) report summary estimates of efficacy based on meta-analysis; 3) identify factors modifying the efficacy of IPC in renal IRI, to inform the design of future clinical trials; and 4) provide insight in the quality of literature in the field of IPC and renal IRI in animal models.

## Analysis

### Literature search strategy, inclusion and exclusion criteria

The present review was based on published results of animal studies on the effects of IPC on renal IRI, which were identified via a systematic computerized search in PubMed and Embase. The inclusion criteria and method of analysis were specified in advance and documented in a protocol. The databases were searched for published articles up to October 19^th^ 2011. The full search strategies for PubMed and EMBASE are included in Appendix S1, and involved the following search components: “animal” [25], [26], “kidney”, “ischemia reperfusion injury” and “preconditioning”. Studies were included in the systematic review if they fulfilled all of the following criteria: 1) the study assessed the effect of remote or local IPC on renal IRI; 2) the study was performed in animals *in vivo*; 3) the study was an original full paper which presented unique data. Studies were excluded if 1) the renal IRI model involved cold storage of the kidney or 2) the study was performed only in genetically modified animals. Study selection was performed independently by two reviewers (TM and KW) on the basis of title and abstract. In case of doubt, the whole publication was evaluated. Differences were clarified by discussion with a third investigator (MW). No language restrictions were applied. If necessary, papers in languages other than English were translated by scientists (native speakers for that particular language) within the Radboud University Nijmegen Medical Centre.

### Study characteristics and data extraction

The following study characteristics and data items were extracted from the studies included: animal species, strain, sex, number of animals in treatment and control groups, measures of randomization, measures of blinding, number of animals excluded for statistical analysis, reason for exclusion of animals, reported outcome measures and results. Bibliographic details such as author, journal, and year of publication were also registered. Three outcome measures were assessed: serum creatinine, BUN and histological renal damage. For histology, data could be extracted if the authors used the Jablonski [27] score for renal damage, or an adapted version of this scoring system.

Data were extracted if raw data or group averages, standard deviation (SD) or standard error (SE), and number of animals per group (n) were reported, or could be recalculated. For 30 articles, relevant outcome measures or study details were not reported. We therefore contacted these authors via e-mail and received response from eight authors, six of which provided additional data. For two papers, authors reported using 6–8 animals per group and we included these data using n = 6 animals [28], [29]. If the number of animals was stated less specific (*e.g.* >3 animals or 4–8 animals), and the exact numbers could not be retrieved by contacting the authors, data were not included. If SE was reported, this was converted to SD for meta-analysis. If a study contained separate groups for each gender, or several preconditioning protocols, these groups were analyzed as if they were separate studies. If two or more identical groups existed, the data were pooled for these groups. If outcomes were measured at several time points, we chose the time point at which efficacy was greatest. In 8 out of 11 cases, this was 24 h post ischemia, which was also the most common time of measurement overall (see Table S1). When data were presented only graphically, we contacted authors to obtain the numerical values. If these were not available, data were measured using digital image analysis software (ImageJ; http://rsbweb.nih.gov/ij/).

### Assessment of methodological quality

We designed a 16-point rating system to assess the methodological quality of the included publications (see Table S2 and legend for details). Concerning the number of excluded animals, we assumed that there had been no exclusion if the number of animals per group mentioned in the materials and methods section was identical to the number stated in the figure legends or results section.

### Data synthesis and statistical analyses

Data were analyzed using Review Manager Version 5.1 (Copenhagen, The Nordic Cochrane Centre, The Cochrane Collaboration, 2011). Meta-analysis was performed for the outcome measures serum creatinine, BUN and histology score, by computing the standardized mean difference (SMD; the mean of the experimental group minus the mean of the control group divided by the pooled SD of the two groups). To account for anticipated heterogeneity, we used random effect models in which some heterogeneity is allowed. Subgroup analyses were pre-defined and performed for all outcome measures, and were used to assess the influence of variables on IPC efficacy, as well as to explore possible causes for heterogeneity. The five subgrouping variables were: timing of index ischemia (late or early window of protection), preconditioning protocol (fractionated or continuous), site of preconditioning (LIPC, RIPC or both), animal species (rat or mouse) and gender (male, female or both). Differences between subgroups were determined by calculating the difference between the respective SMDs (ΔSMD) and confidence interval (CI) of the difference. Furthermore, subgroup interaction analysis was performed in an attempt to further explain the expected study heterogeneity: we compared smaller sets of experiments by combining two subgrouping variables, *e.g.* early-RIPC *vs.* early-LIPC. Unless indicate otherwise, data are presented as SMD and 95% CIs.

For each outcome measure, we assessed the possibility of publication bias by visually evaluating the possible asymmetry in funnel plots. Finally, we investigated whether study methodology influenced the results of our meta-analysis. Pre-specified sensitivity analysis was performed to assess whether the chosen cut-off point for early *vs.* late window of protection influenced the outcome of this subgroup analysis.

## Results

### Study selection and characteristics

**Figure 1:**
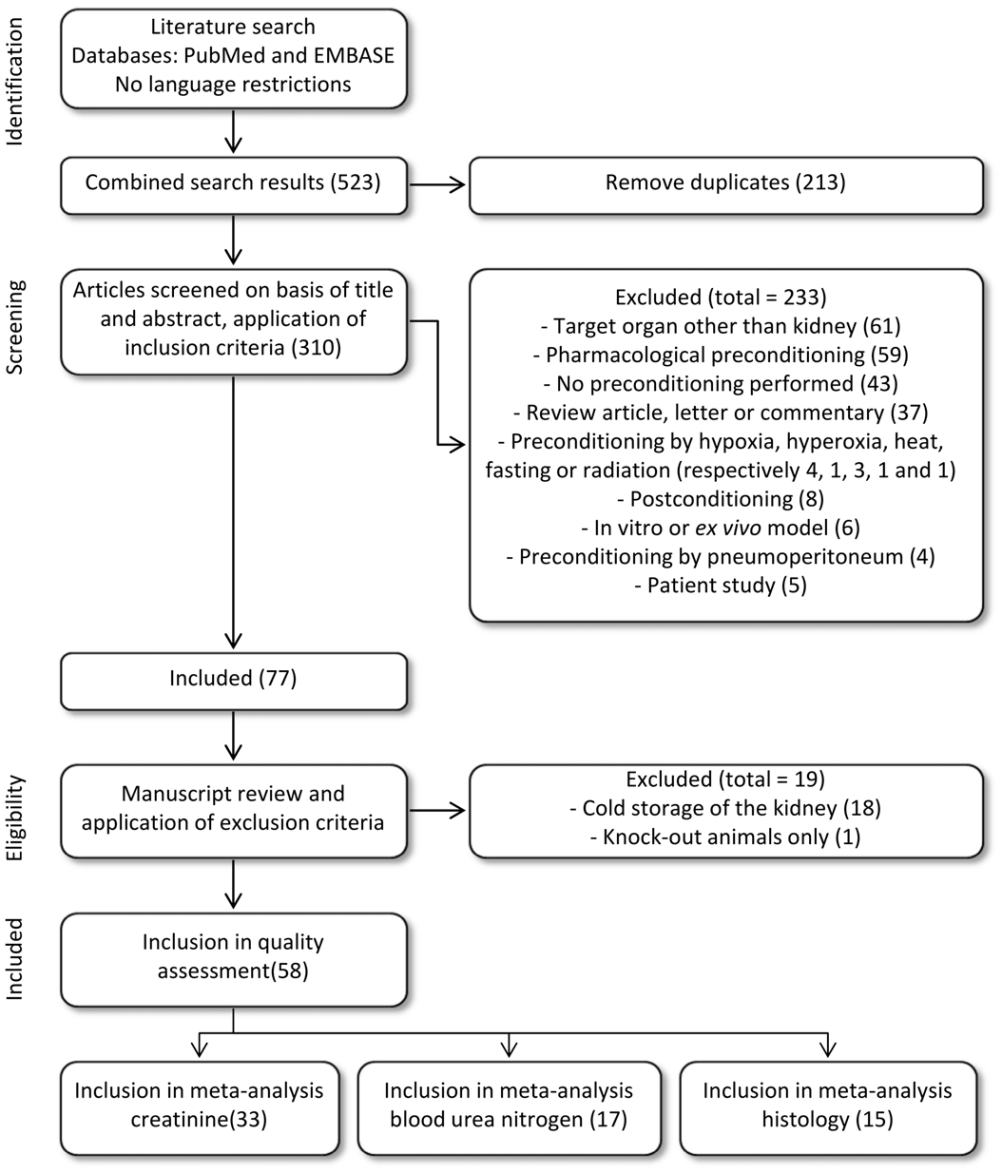
Flow chart of study selection. The number of studies in each phase is indicated between brackets.

The electronic search strategy retrieved 253 records from PubMed and 270 articles from EMBASE, 310 of which were unique. Seventy-seven papers met our inclusion criteria. On the basis of predefined criteria, 19 reports were excluded and the remaining 58 articles were retrieved in full (see Figure 1).

The characteristics of the included studies are summarized in Table S1. There was a large variation in study characteristics. In 76% of the 58 included studies, the delay between the preconditioning stimulus and the index ischemia was <24 h, which we considered to be the early window of protection. Eleven studies (19%) assessed protection in the late window of protection (timing of index ischemia ≥24 h after IPC), and 3 studies (5%) reported data for both late and early window(s) of protection. For the early window of protection, the delay between IPC and index ischemia was 4 to 40 min (average 9±7 min). For the late window of protection, this was 24 h up to 12 wk (average 17±23 d). In 28 of the 58 studies (48%), the ischemic preconditioning protocol consisted of one continuous stimulus. Twenty-two studies (38%) used only fractionated protocol(s), i.e. 2 to 5 cycles of brief ischemia and reperfusion, whereas 8 studies employed both fractionated and continuous stimuli. The majority of studies focussed on the protective effects of LIPC on renal IRI. However, 5 studies assessed the effects of RIPC, using hind limb, intestine, liver or subphrenic aortic occlusion as remote stimuli. In 4 studies, both LIPC and RIPC of one kidney to its contralateral counterpart were performed (either intentionally, or as a result of a bilateral preconditioning stimulus and a unilateral index ischemia). Out of all 58 included studies, 14 were conducted in mice (24%), 34 in rat (59%), and 10 in other species, namely rabbit (7%), dog (5%) and pig (5%). Eight out of 58 studies (14%) were performed in female animals, 37 in males (64%), and 4 studies used animals of both genders (7%). Nine studies did not report the gender of the animals.

### Methodological quality of studies

**Figure 2:**
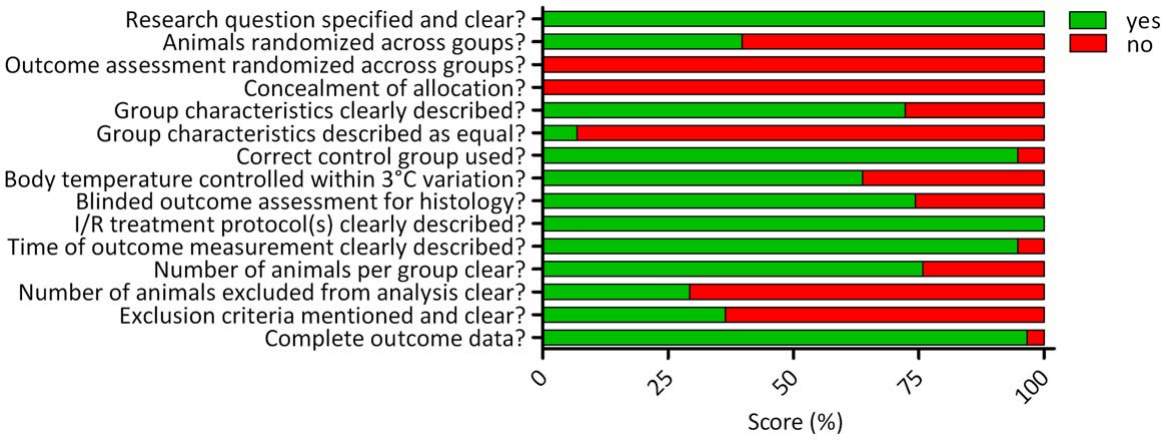
Quality assessment score, averaged per item. Many studies scored poorly on key characteristics of scientific practice, and measures to avoid bias, such as characteristics of the subject population, randomization, blinding and exclusion criteria.

The results of the quality assessment of the 58 studies included in this systematic review are shown in Table S2 and Figure 2. On average, studies reported 9 out of 16 characteristics (59±10%). The lowest score was 5 out of 15 items (33%), and the highest scoring studies reported 12 items out of 14 (80%). In the quality assessment of clinical trials, randomization, blinding, and description of withdrawals are key quality measures. However, only 40% of the animal studies included in this systematic review reported randomization of the animals across treatment groups. Out of the 39 studies in which renal histology was an outcome measure, 74% reported blinding of the outcome assessment. No study reported blinding for any other outcome measure. Only 29% of the studies reported the number of animals excluded, 64% of which did not state the reason for exclusion. Although the strong influence of body temperature on renal damage has been shown in both large and small animal models, 36% of the studies did not report whether the body temperature of the animals was controlled.

### Meta analysis of outcome measures

**Figure 3:**
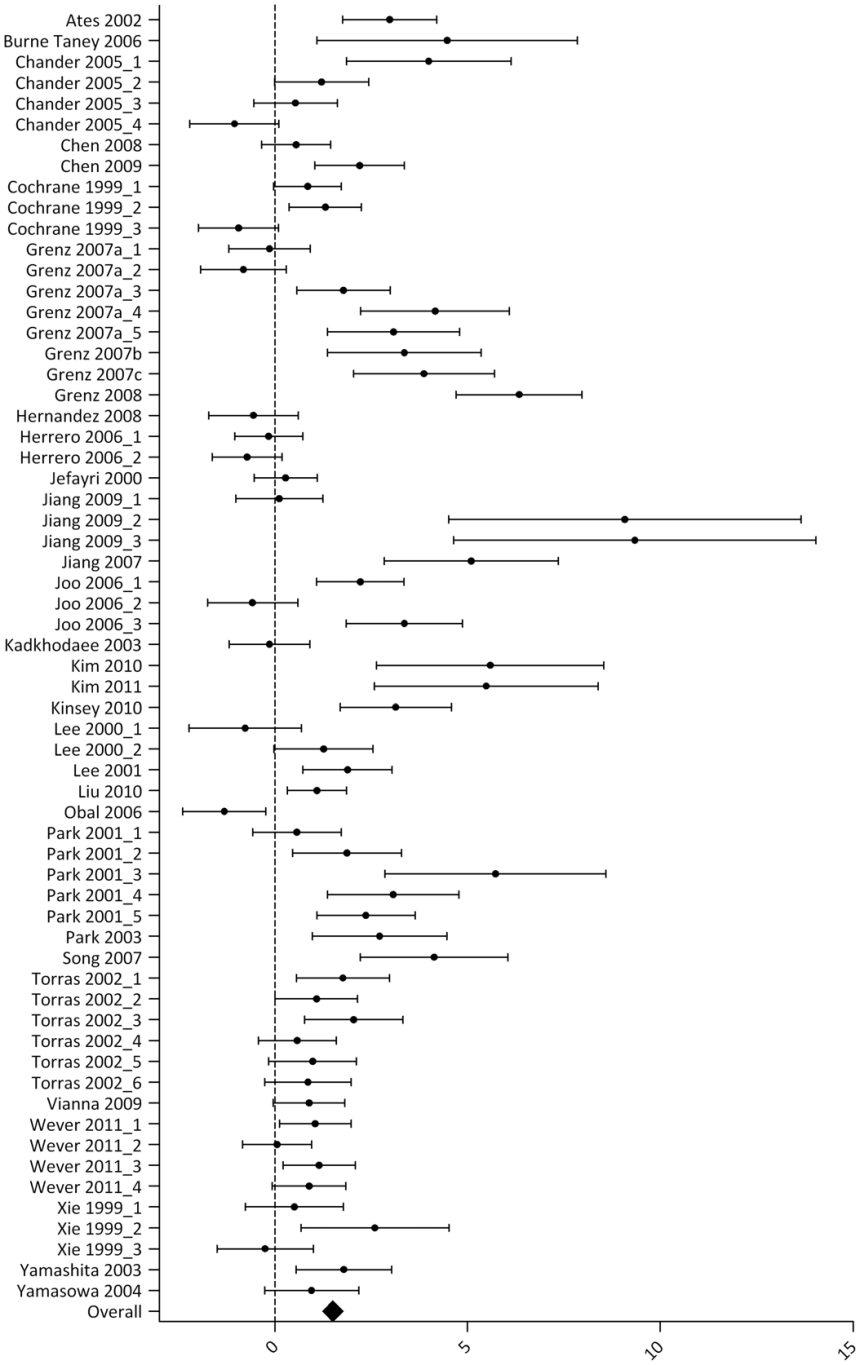
Effect of IPC on serum creatinine after renal IRI. Left side favours control (renal IRI only), right side favours IPC. An overall beneficial effect of IPC on serum creatinine was observed (1.54 [1.16, 1.93]). Data presented as SMD and 95% CI.

Results for the outcome measure serum creatinine are summarized in Table S3 and Figure 3. Thirty-two articles studied the effect of one or more IPC protocols on serum creatinine after renal IRI. The analysis contained 62 experiments, including data for 512 control animals which underwent renal IRI only, and 492 animals that underwent IPC + renal IRI. In 36 experiments, the SMD and 95% CI indicated that IPC significantly reduced the IRI-induced rise in serum creatinine. One study reported a negative effect of IPC on serum creatinine [30]. Overall analysis showed that IPC reduced serum creatinine after IRI (1.54 [1.16, 1.93], p<0.00001). Overall study heterogeneity was high (83%).

Subgroup analysis showed a beneficial effect of IPC for all subgroups, except for female (notably, this subgroup contained only two experiments and was therefore excluded from further statistical analysis). We also found a subgroup effect of the variable ‘window of protection’ (Table S2, filled squares). The ΔSMD and CI of the difference for early *vs.* late was 2.43 [1.29, 3.57], indicating that the late window of protection of IPC was more effective in reducing serum creatinine than the early window. Subgroup analysis indicated a higher IPC efficacy in studies conducted in mouse *vs.* rat (Table S2, triangles; ΔSMD 1.7 [1.5, 1.90]). For other species (dog, pig, rabbit) subgroups were too small to perform reliable subgroup analysis. No difference in IPC efficacy was observed for continuous *vs.* fractionated; ΔSMD 0.46 [−0.30, 1.22]), or males only *vs.* groups of mixed gender (ΔSMD 0.38 [−0.60, 1.36]). For site of preconditioning, no differences were found when comparing the subgroups LIPC *vs.* RIPC (ΔSMD 0.06 [−0.98, 1.10]), LIPC *vs*. LIPC +RIPC (ΔSMD 1.01 [−0.44, 2.46]) or RIPC *vs*. LIPC+RIPC (ΔSMD 0.95 [−0.73, 2.63]).

**Figure 4:**
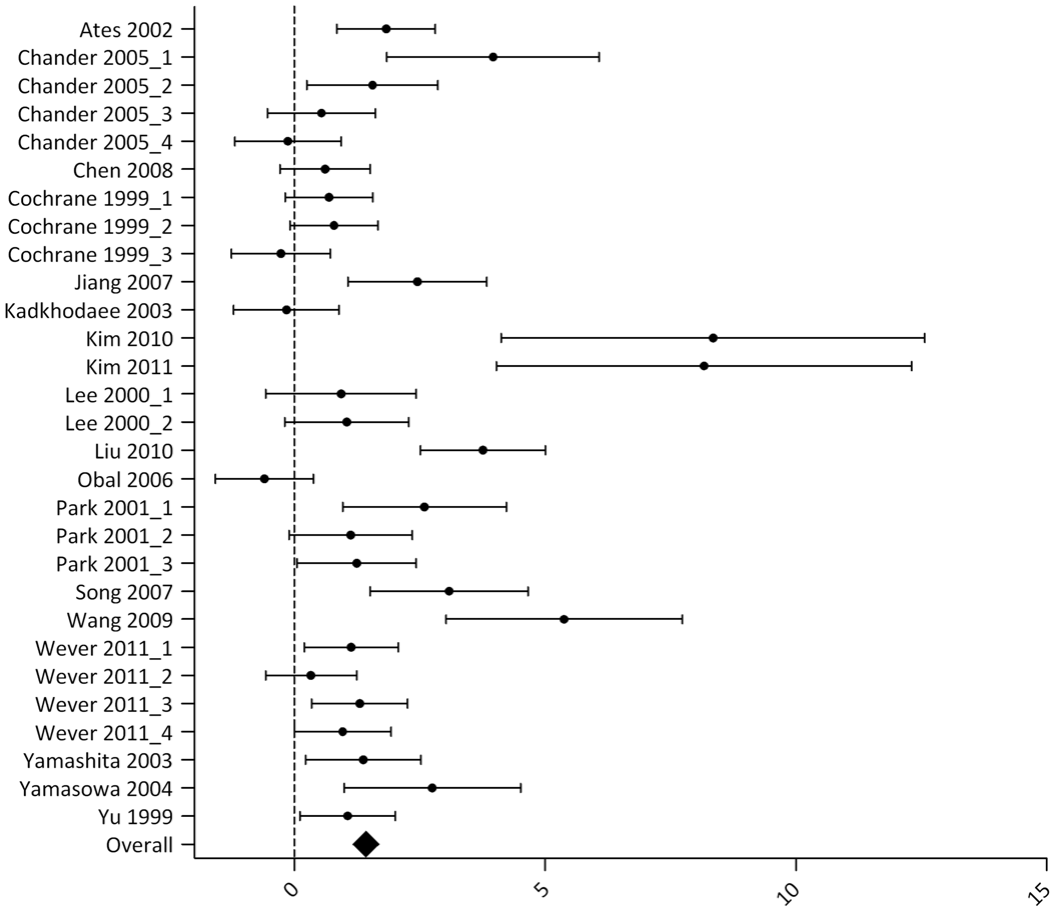
Effect of IPC on BUN after renal IRI. Left side favours control (renal IRI only), right side favours IPC. An overall beneficial effect of IPC on BUN was observed (1.42 [0.97, 1.87]). Data presented as SMD and 95% CI.

Results for the outcome measure BUN are summarized in Table S4 and Figure 4. Seventeen articles studied the effect of one or more IPC protocols on BUN after renal IRI. In 20 out of 29 experiments, the IRI-induced rise in BUN was significantly reduced in animals undergoing IPC, when compared to a control group that underwent IRI only (overall effect size 1.42 [0.97, 1.87]; p<0.00001). Overall study heterogeneity was high (76%).

Subgroup analysis showed that the beneficial effect of IPC on BUN was present in all subgroups. Between-subgroup analysis revealed a higher IPC efficacy in mouse *vs.* rat (Table S3, triangles; ΔSMD 2.12 [0.48, 3.76). No effect was found for the window of protection (early *vs*. late; ΔSMD 1.25 [−0.05, 2.55]) or the IPC protocol (continuous *vs.* fractionated; ΔSMD 0.96 [−0.03, 1.95]). Furthermore, the site of preconditioning did not influence IPC efficacy: LIPC *vs.* RIPC, LIPC *vs.* LIPC +RIPC and RIPC *vs.* LIPC+RIPC, respectively ΔSMD 0.2 [−0.69, 1.09]), ΔSMD 0 [−1.03, 1.03] and ΔSMD 0.2 [−0.82, 1.22]. Subgroup analysis could not be performed for the variable ‘gender’, because of insufficient data.

**Figure 5:**
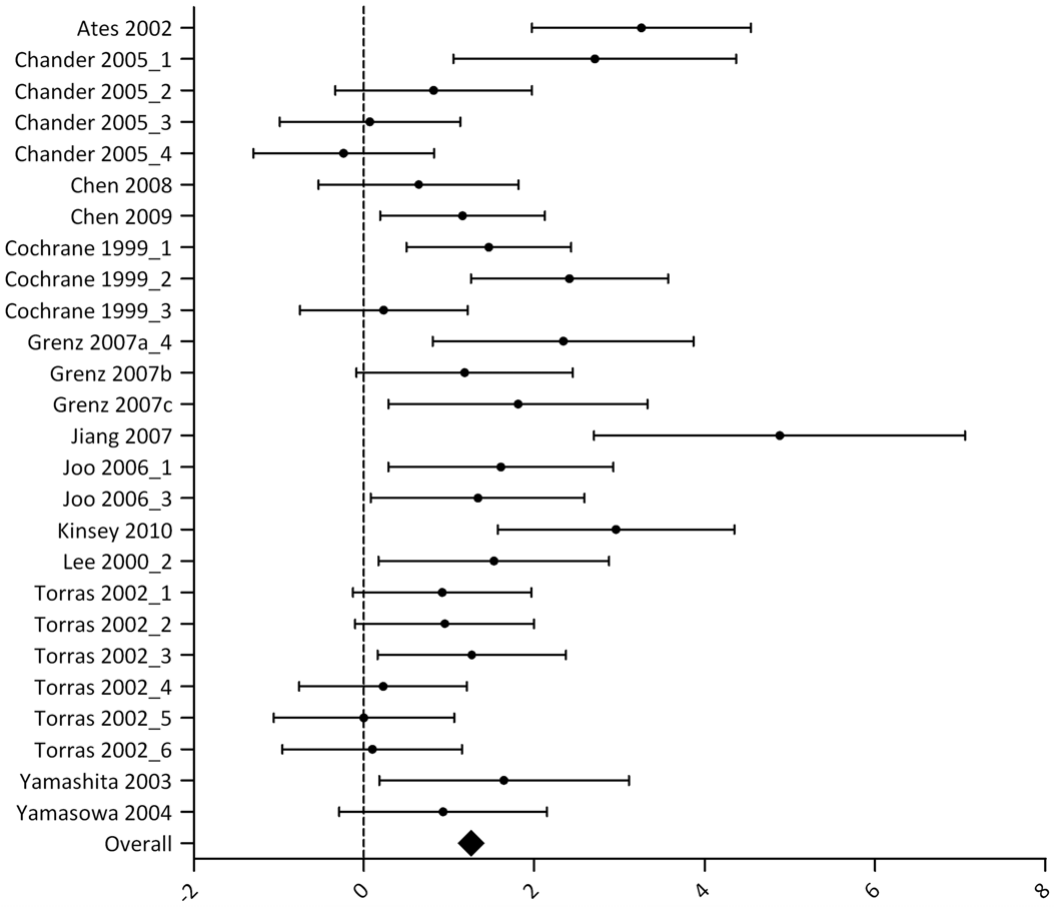
Effect of IPC on renal histology after renal IRI. Left side favours control (renal IRI only), right side favours IPC. An overall beneficial effect of IPC on renal histology was observed (1.22 [0.89, 1.35]). Data presented as SMD and 95% CI.

Results for the outcome measure renal histology are summarized in Table S5 and Figure 5. Twenty-six experiments from 15 studies reported the effect of IPC on the Jablonski score for renal histology. Eight studies using a histology score not comparable with Jablonski's were excluded from analysis. Data included contained 205 control and 191 IPC-treated animals. Overall, IPC had a significant renal protective effect of 1.12 [0.89, 1.35]. Overall study heterogeneity was 63%.

Subgroup analysis showed that the beneficial effect of IPC on histology was present in all subgroups. Between-subgroup analysis could only be performed for the variables window of protection, IPC protocol, gender and animal species, because of insufficient numbers of experiments in the other subgroups. No significant differences between subgroups were found (early *vs*. late, ΔSMD 1.8 [−0.07, 3.67]; continuous *vs.* fractionated, ΔSMD 0.3 [−0.50, 1.10]; males *vs.* mixed gender, ΔSMD 0.25 [−0.58, 1.08]; rat *vs.* mice, ΔSMD 0.55 [−0.14, 1.24]).

### Subgroup interaction analysis

In an attempt to further explain the expected study heterogeneity, subgroup interaction analysis was performed for all subgroup interactions which contained three or more experiments. Study heterogeneity was not notably reduced by combining subgroup variables and remained on average 80±6% for serum creatinine, 62±23% for BUN and 47±30% for renal histology. The analyses revealed no significant differences in the interaction between subgroups, and did therefore not alter the results of the subgroups analysis. Interestingly, for serum creatinine, the subgroup interactions early-RIPC and continuous-RIPC did not show an overall effect of IPC, whereas early-LIPC and continuous-LIPC did show the protective effect. This may indicate that the positive effect of an early window of protection, or the benefits of a continuous IPC protocol are less pronounced for RIPC than for LIPC. However, because of the small number of experiments in these subgroups interactions (six and three experiments, respectively), these results must be interpreted with care.

### Publication bias

The presence of publication bias was assessed for all outcome measures. Visual analysis of funnel plots revealed that small, negative studies appeared to be underrepresented (data not shown). This was especially true for serum creatinine and BUN, and to a lesser extent for renal histology data.

### Sensitivity analysis

To assess the robustness of our findings, we undertook a sensitivity analysis by redefining the cut off-point for the early window of protection at a later time point (<48 h) or an earlier time point (<6 h). This did not significantly alter the outcome of any of the outcome measures (data not shown).

## Discussion

Here we report a unique systematic review and meta-analysis of current literature reporting experimental animal models of IPC in renal IRI. Three important outcome measures were assessed, namely serum creatinine, BUN and histological renal damage quantified by Jablonski score. For all three, protective effects of IPC were observed, *i.e.* IPC reduced serum creatinine (1.54 [1.16, 1.93]), BUN (1.42 [0.97, 1.87]) and histological damage (1.12 [0.89, 1.35]) after IRI, when compared to control animals undergoing renal IRI only. Importantly, in the clinical setting, serum creatinine currently remains the gold standard to assess renal function. In rodents however, questions have been raised regarding the reliability of creatinine for measuring renal function, since the impact of tubular creatinine excretion on creatinine clearance is even larger in mice than in humans [31]. We therefore put forward that other outcome measures, such as BUN and/or renal histology may also be of great value when translating animal study results to the human setting. Furthermore, other renal damage markers such as Kidney Injury Molecule-1 (KIM-1) and Neutrophil Gelatinase-Associated Lipocalin (NGAL) are gaining ground in clinical practice [32]. Reporting these markers in both animal and human studies may provide further information for the translation of animal study data to the human setting.

We performed subgroup analysis to investigate several pre-defined factors which we hypothesized to modify the efficacy of IPC in renal IRI, namely: window of protection (early or late), IPC protocol (continuous or fractionated), site of preconditioning (RIPC, LIPC or both), species (mouse or rat) and gender (male, female or mixed). The protective effects of IPC were persistent in all subgroups, for all outcome measures, except for female (only 2 experiments). Based on the latter observation, we propose the need for future studies should in females, since it has been shown that there is a significant difference between males and females for cardiac IPC efficacy (*e.g.* [33]).

For serum creatinine, the window of protection influenced the efficacy of IPC: IPC was more effective when conducted >24 h before index ischemia (late window of protection), as compared to an early widow of protection (<24 h before index ischemia). We observed the same trend towards higher efficacy in the late window of protection for BUN and renal histology. The cut-off point of <24 h for the early window could be redefined at <6 h or <48 h without significantly influencing these results, since the vast majority of experiments (93%) investigated a time window of either <40 minutes, or >4 days. The remaining 7% of the experiments concerned a time window of 6–24 h between IPC and IRI. Thus, there is a large gap in these data which makes it difficult to assess the optimal window of protection for IPC. Nevertheless, our data strongly indicate that the late window of protection might be more effective to reduce renal IRI as compared to the early window. This finding is particularly interesting since almost all clinical trials currently registered at Clinicaltrials.gov investigating the effects of LIPC and RIPC use only the early window of protection. To our knowledge data on the efficacy of combined activation of the early and late window in humans is lacking.

The second variable which influenced IPC efficacy was animal species: for serum creatinine and BUN data, IPC was more effective when performed in mice *vs.* rats. This suggests that mouse models of renal IPC may be more sensitive when compared to rat, and are thus the preferable models for future animal studies. Furthermore, this finding implicates that IPC efficacy is species-specific, and therefore the protective effect may be greater, or less pronounced in large animals and humans. This illustrates the difficulty in directly translating results from animal studies to the human setting, and further studies in large animals and humans are necessary to clarify this issue.

No significant differences were observed for the variables IPC protocol (continuous *vs.* fractionated) or site of preconditioning (LIPC, RIPC or both). The latter finding is interesting, since the use of LIPC in clinical practice is limited because of the risk of damage to major vascular structures, and the fact that even brief ischemia may damage the target organ in vulnerable patients. RIPC therefore has more potential for clinical application, since the IPC stimulus can be applied to *e.g.* a limb, which is easy to handle and relatively resistant to IRI. Our finding that RIPC and LIPC are equally effective indicates that RIPC has an at least equal potential for translation to the clinic, although it must be noted that only two studies used the limb as remote organ. Subgroup analysis of the serum creatinine levels in animals undergoing simultaneous LIPC of one kidney and RIPC of the contralateral kidney show a trend towards higher efficacy (Table S2, filled circles), indicating that a combination of LIPC and RIPC may have an additive effect. However, this result must be interpreted with care, because of the low number of experiments included.

### Methodological quality of studies

Our assessment reveals that there is much to gain in terms of the methodological quality of animals studies in this field. Key characteristics of scientific practice, and measures to avoid bias, such as characteristics of the subject population, randomization, blinding and exclusion criteria, were infrequently reported. A number of recent systematic reviews show that this is the case in many fields of animals research. For scientific and ethical reasons, it is urgent that the standards routinely applied in human research become standard of practice in animal research as well. While it is possible that some authors merely failed to report these details, there is reason for concern, since it is unclear whether there is a significant difference between the reported study quality and the actual study quality. For this reason, better reporting of animal studies is crucial. Regrettably, there appears to be an inverse correlation between the impact factor of the journal in which the study is published, and the required detail of the materials and methods description [24]. The high heterogeneity of the data presented in this systematic review may be explained in part by the differences in study quality, as well as the lack of consensus and general standards of practice in animal studies. It has proven difficult to obtain missing data by contacting authors directly, which further emphasizes the importance of adequately reporting animal studies. However, in spite of insufficient reporting, systematic review and meta-analysis of current publications aid in making possible bias transparent, and can provide us with valuable new insights, which will support the translation of animal data to the clinical setting.

### Strengths and limitations

The major strength of our study is that as far as we are aware, we are the first performing a systematic review and meta-analysis on renal protection by IPC in animal studies. We were able to include a large number of studies per outcome measure, which enabled us to investigate the effect of several subgroup variables.

Some potential limitations should be discussed. Firstly, the extracted data are highly heterogeneous, which is most likely due to a large variety in experimental designs used and the variation in study quality. The fact that our subgroup interaction analysis did not notably reduce heterogeneity supports this notion. Although we have tried to account for this heterogeneity by using a random effects model and performing subgroup and sensitivity analysis, pooling of the results may not be appropriate for all subgroups. Therefore, differences between (small) subgroups should be interpreted with caution and be used to generate new hypotheses, rather than for drawing final conclusions. However, all studies provide information on the association between IPC and IRI in the animal kidney, and are thus valuable for this systematic review.

Secondly, the included studies may be subject to publication bias. Visual analysis of funnel plots revealed that only small, negative studies appeared to be underrepresented in current literature on IPC in renal IRI. Asymmetry was most notable in serum creatinine and BUN data. This may indicate that publication bias is present, which could cause overestimation of the effect sizes. Importantly, funnel plot asymmetry can result from non-publication of negative results, but may also be caused by other factors, such as true study heterogeneity, or differences in study quality [34]. Our finding that the study quality is rather heterogeneous may therefore explain part of the funnel plot asymmetry.

### Clinical implications

Both LIPC and RIPC (and also the combination of the two), appear to have the potential to reduce IRI, and since RIPC by brief limb ischemia has the advantage of being safe and easy to perform, the latter has the greatest potential for clinical practice. In contrast to the variety of IPC protocols used in animal studies, current clinical trials on RIPC in renal IRI are using similar preconditioning protocols, namely fractionated IPC stimuli, and a short delay between IPC and index ischemia (early window of protection). The current review indicates that even though this approach might be effective, efficacy could be even higher in the late window of protection. Future studies should be designed to investigate the optimal window of protection in patients, taking into account the possible difference between acute and delayed ischemic preconditioning. Whether a combination of the two is additive or even synergistic requires further testing in animal and human models as well.

It is important to realize that, to date, no studies (animal or human) have investigated the effect of co-medication and co-morbidities such as diabetes, hypertension or obesity, on IPC in renal IRI. For the heart, it has been shown that medication and co-morbidities influence IPC efficacy (reviewed e.g. in [35]). Similarly, differences in IPC efficacy between genders may indicate that the optimal IPC stimulus is different in males *vs.* females. We propose that future clinical studies should be designed to optimize IPC efficacy for certain patient groups, and that animal studies in this area can inform the design of such clinical trials. Furthermore, a better mechanistic insight is needed in the cause of the observed interspecies difference. These data will give us a clue whether translation to humans is feasible.

### Conclusion

The currently applied approach of systematic review and meta-analysis indicates that, in animal studies, IPC has an overall protective effect on the kidney, since it reduces serum creatinine, blood urea nitrogen (BUN) and renal damage as assessed by histology after IRI. We found that IPC is more effective in reducing serum creatinine when the IPC stimulus is applied >24 h before index ischemia (late window of protection), a trend which was also observed for BUN and renal histology data. Furthermore, serum creatinine and BUN data showed an effect of animal species on IPC efficacy: IPC was more effective when performed in mice *vs.* rats. No significant differences were observed for the variables site of preconditioning (local, remote or both) or IPC protocol (continuous *vs.* fractionated). Our review indicates that current clinical trials on RIPC may not be optimally designed, and further optimization may be necessary for successful translation to the clinical setting.

## Supporting Information

Table S1 Study characteristics.
(DOC)

Table S2 Methodological quality.
(DOC)

Table S3 Subgroup analysis serum creatinine.
(DOC)

Table S4 Subgroup analysis blood urea nitrogen.
(DOC)

Table S5 Subgroup analysis histology.
(DOC)

Appendix S1 Full search strategy for PubMed and EMBASE.
(DOC)
