# Supplementary material for: Ischemic Preconditioning in the Animal Kidney, a Systematic Review and Meta-Analysis
Source: PLoS One. 2012 Feb 28;7(2):e32296. doi: 10.1371/journal.pone.0032296 (PMC3289650; doi:10.1371/journal.pone.0032296)
Supplement: Table S2 — Methodological quality. (DOC) [file pone.0032296.s002.doc]

| **Table S2|Methodological quality** | | | | | | | | | | | | | | | | | | | | | | | | | | | | | |
| --- | --- | --- | --- | --- | --- | --- | --- | --- | --- | --- | --- | --- | --- | --- | --- | --- | --- | --- | --- | --- | --- | --- | --- | --- | --- | --- | --- | --- | --- |
|  | Ateş, 2002 [1] | Aufricht, 2002 [2] | Ayupova, 2009 [3] | Burne-Taney, 2006 [4] | Cao, 2010 [5] | Chander, 2005 [6] | Chen, 2008 [7] | Chen, 2009 [8] | Cochrane, 1999 [9] | Grenz, 2007a [10] | Grenz, 2007b [11] | Grenz, 2007c [12] | Grenz, 2008 [13] | Guye, 2010 [14] | Hernandez, 2008 [15] | Herrero, 2006 [16] | Hyodo, 2009 [17] | Islam, 1997 [18] | Jang, 2008 [19] | Jefayri, 2000 [20] | Jiang, 2007 [21] | Jiang, 2009 [22] | Joo, 2006 [23] | Kadkhodaee, 2004 [24] | Kim, 2010 [25] | Kim, 2011 [26] | Kinsey, 2010 [27] | Kosieradzki, 2003 [28] | Lazaris, 2009 [29] |
| Research question specified and clear? | + | + | + | + | + | + | + | + | + | + | + | + | + | + | + | + | + | + | + | + | + | + | + | + | + | + | + | + | + |
| Animals randomized across groups? | - | - | - | - | - | - | - | + | + | - | - | - | - | + | - | - | + | + | - | + | + | + | - | - | - | + | - | - | - |
| Outcome assessment randomized across groups? | - | - | - | - | - | - | - | - | - | - | - | - | - | - | - | - | - | - | - | - | - | - | - | - | - | - | - | - | - |
| Concealment of allocation? | - | - | - | - | - | - | - | - | - | - | - | - | - | - | - | - | - | - | - | - | - | - | - | - | - | - | - | - | - |
| Group characteristics clearly described?* | + | +^†^ | +^†^ | - | +^†^ | +^†^ | + | +^†^ | + | - | - | - | - | + | - | - | +^†^ | + | +^†^ | +^†^ | +^†^ | +^†^ | - | +^†^ | + | +^†^ | + | + | + |
| Group characteristics described as equal? | - | - | - | - | - | - | - | - | - | + | + | + | + | - | - | - | - | - | - | - | - | - | - | - | - | - | - | - | - |
| Correct control group used? | + | + | - | + | + | + | + | + | + | + | + | + | - | + | + | + | + | + | + | + | + | + | + | + | + | + | + | + | + |
| Body temperature controlled within 3°C variation? | - | + | - | + | + | + | + | - | + | + | + | + | + | + | - | - | - | + | + | + | + | + | + | - | + | + | + | + | - |
| Blinded outcome assessment for histology? | + | NA | NA | + | + | + | + | - | + | + | + | + | + | - | + | NA | NA | + | - | - | + | + | + | + | - | NA | - | NA | NA |
| I/R treatment protocol(s) clearly described?^‡^ | + | + | + | + | + | + | + | + | + | + | + | + | + | + | + | + | + | + | + | + | + | + | + | + | + | + | + | + | + |
| Time of outcome measurement clearly described? | + | + | + | + | + | + | + | + | + | + | + | + | + | + | - | + | + | + | + | + | + | + | + | + | + | + | + | + | + |
| Number of animals per group clear? | + | - | - | - | + | + | + | + | + | - | - | - | - | + | + | - | + | + | - | + | + | + | + | + | - | + | - | + | + |
| Number of animals excluded from analysis clear? | - | - | - | - | + | + | - | - | - | + | - | + | - | + | + | - | + | - | - | + | - | - | - | - | - | - | - | + | - |
| Exclusion criteria mentioned and clear? | NA | NA | NA | NA | NA | + | - | - | - | NA | - | NA | - | + | + | NA | NA | - | NA | + | - | - | + | NA | - | NA | NA | + | NA |
| Complete outcome data? | - | + | + | + | + | + | + | + | + | + | + | + | + | - | + | +^§^ | + | + | + | + | + | + | + | + | + | + | + | + | + |
| Total score | 7 | 8 | 6 | 7 | 10 | 11 | 9 | 8 | 10 | 9 | 8 | 9 | 7 | 10 | 8 | 5 | 9 | 10 | 7 | 11 | 10 | 10 | 9 | 8 | 7 | 9 | 7 | 10 | 7 |
| Maximal possible score | 14 | 13 | 13 | 14 | 14 | 15 | 15 | 15 | 15 | 14 | 15 | 14 | 15 | 15 | 15 | 13 | 13 | 15 | 14 | 15 | 15 | 15 | 15 | 14 | 15 | 13 | 14 | 14 | 13 |
| Quality (%) | 50 | 62 | 46 | 50 | 71 | 73 | 60 | 53 | 67 | 64 | 53 | 64 | 47 | 67 | 53 | 38 | 69 | 67 | 50 | 73 | 67 | 67 | 60 | 57 | 47 | 69 | 50 | 71 | 54 |

| **Table S2 (continued)** | | | | | | | | | | | | | | | | | | | | | | | | | | | | | | |
| --- | --- | --- | --- | --- | --- | --- | --- | --- | --- | --- | --- | --- | --- | --- | --- | --- | --- | --- | --- | --- | --- | --- | --- | --- | --- | --- | --- | --- | --- | --- |
|  | Lee, 2000 [30] | Lee, 2001 [31] | Li, 2005 [32] | Liu, 2010 [33] | Mahfoudh-Boussaid, 2007 [34] | Obal, 2006 [35] | Ogawa, 2000 [36] | Ogawa, 2001 [37] | Ogawa, 2002 [36] | Orvieto, 2007 [38] | Park, 2001 [39] | Park, 2003 [40] | Patshan, 2006 [41] | Salehipour, 2007 [42] | Sola, 2003 [43] | Song, 2007 [44] | Sugino, 2001 [45] | Timsit, 2008 [46] | Toosy, 1999 [47] | Torras, 2002 [48] | Treska, 2006 [49] | Vianna, 2009 [50] | Wang, 2009 [51] | Wever, 2011[52] | Wu, 2009 [53] | Xie, 1999 [54] | Yamashita, 2003 [55] | Yamasowa, 2005 [56] | Yu, 1999 [57] | Overall score (%) |
| Research question specified and clear? | + | + | + | + | + | + | + | + | + | + | + | + | + | + | + | + | + | + | + | + | + | + | + | + | + | + | + | + | + | 100 |
| Animals randomized across groups? | + | - | + | + | + | + | - | - | - | + | - | - | - | + | - | + | - | - | + | - | - | + | + | + | - | + | - | - | + | 40 |
| Outcome assessment randomized across groups? | - | - | - | - | - | - | - | - | - | - | - | - | - | - | - | - | - | - | - | - | - | - | - | - | - | - | - | - | - | 0 |
| Concealment of allocation? | - | - | - | - | - | - | - | - | - | - | - | - | - | - | - | - | - | - | - | - | - | - | - | - | - | - | - | - | - | 0 |
| Group characteristics clearly described?* | + | +^†^ | +^†^ | +^†^ | +^†^ | +^†^ | +^†^ | +^†^ | +^†^ | - | +^†^ | +^†^ | - | - | +^†^ | +^†^ | +^†^ | +^†^ | +^†^ | +^†^ | +^†^ | - | - | +^†^ | +^†^ | +^†^ | + | - | + | 76 |
| Group characteristics described as equal? | - | - | - | - | - | - | - | - | - | - | - | - | - | - | - | - | - | - | - | - | - | - | - | - | - | - | - | - | - | 7 |
| Correct control group used? | + | + | - | + | + | + | + | + | + | + | + | + | + | + | + | + | + | + | + | + | + | + | + | + | + | + | + | + | + | 95 |
| Body temperature controlled within 3°C variation? | + | + | - | + | + | + | - | - | + | + | + | + | - | - | + | + | - | - | + | + | - | + | - | + | - | - | - | - | + | 64 |
| Blinded outcome assessment for histology? | + | NA | NA | + | NA | + | NA | NA | NA | NA | - | NA | NA | - | NA | + | + | + | - | + | + | + | NA | + | NA | - | + | + | + | 74 |
| I/R treatment protocol(s) clearly described?^‡^ | + | + | + | + | + | + | + | + | + | + | + | + | + | + | + | + | + | + | + | + | + | + | + | + | + | + | + | + | + | 100 |
| Time of outcome measurement clearly described? | + | + | + | + | + | + | + | + | + | + | + | + | + | + | + | + | + | + | + | + | + | + | - | + | + | - | + | + | + | 95 |
| Number of animals per group clear? | + | + | + | + | + | + | + | + | + | + | + | - | - | + | - | + | + | + | + | + | + | + | + | + | + | + | + | + | + | 76 |
| Number of animals excluded from analysis clear? | - | - | - | - | - | + | - | - | - | - | - | - | - | - | - | + | + | + | - | - | - | - | + | + | - | + | - | - | + | 29 |
| Exclusion criteria mentioned and clear? | - | NA | + | + | NA | + | - | NA | NA | + | - | - | - | NA | - | - | NA | NA | + | - | - | - | NA | NA | - | NA | - | + | NA | 36 |
| Complete outcome data? | + | + | + | + | + | + | + | + | + | + | + | + | + | + | + | + | + | + | + | + | + | + | + | + | + | - | + | + | - | 97 |
| Total score | 10 | 8 | 8 | 11 | 9 | 12 | 7 | 7 | 8 | 9 | 8 | 7 | 5 | 7 | 7 | 11 | 9 | 9 | 10 | 9 | 8 | 9 | 7 | 11 | 7 | 7 | 8 | 9 | 10 | **8** |
| Maximal possible score | 15 | 13 | 14 | 15 | 13 | 15 | 14 | 13 | 13 | 14 | 15 | 14 | 14 | 14 | 14 | 15 | 14 | 14 | 15 | 15 | 15 | 15 | 13 | 14 | 14 | 14 | 15 | 15 | 14 | **14** |
| Quality (%) | 67 | 62 | 57 | 73 | 69 | 80 | 50 | 54 | 62 | 64 | 53 | 50 | 36 | 50 | 50 | 73 | 64 | 64 | 67 | 60 | 53 | 60 | 54 | 79 | 50 | 50 | 53 | 60 | 71 | **59** |
| 1 = yes, 0 = no, NA = not applicable; *required are: species, strain, sex, and weight or age; ^†^weight or age missing; ^‡^required are: number and duration of preconditioning ischemic period(s), number and duration of preconditioning reperfusion period(s), timing and duration of index ischemia; ^§^no, but explained | | | | | | | | | | | | | | | | | | | | | | | | | | | | | | |

**References**

1. Ateş E, Genç E, Erkasap N, Erkasap S, Akman S, et al. (2002) Renal protection by brief liver ischemia in rats. Transplantation 74: 1247–1251. doi:10.1097/01.TP.0000032752.61372.36.

2. Aufricht C, Bidmon B, Ruffingshofer D, Regele H, Herkner K, et al. (2002) Ischemic conditioning prevents Na,K-ATPase dissociation from the cytoskeletal cellular fraction after repeat renal ischemia in rats. Pediatr Res 51: 722–727.

3. Ayupova DA, Singh M, Leonard EC, Basile DP, Lee BS (2009) Expression of the RNA-stabilizing protein HuR in ischemia-reperfusion injury of rat kidney. Am J Physiol Renal Physiol 297: F95–F105. doi:10.1152/ajprenal.90632.2008.

4. Burne-Taney MJ, Liu M, Baldwin WM, Racusen L, Rabb H (2006) Decreased capacity of immune cells to cause tissue injury mediates kidney ischemic preconditioning. J Immunol 176: 7015–7020.

5. Cao C, Wang S, Fan L, Wan X, Liu X, et al. (2010) Renal protection by ischemic preconditioning is associated with p50/p50 homodimers. Am J Nephrol 31: 1–8. doi:10.1159/000252844.

6. Chander V, Chopra K (2005) Role of nitric oxide in resveratrol-induced renal protective effects of ischemic preconditioning. J Vasc Surg 42: 1198–1205. doi:10.1016/j.jvs.2005.08.032.

7. Chen H, Xing B, Liu X, Zhan B, Zhou J, et al. (2008) Similarities between ozone oxidative preconditioning and ischemic preconditioning in renal ischemia/reperfusion injury. Arch Med Res 39: 169–178. doi:10.1016/j.arcmed.2007.09.005.

8. Chen X, Liu X, Wan X, Wu Y, Chen Y, et al. (2009) Ischemic preconditioning attenuates renal ischemia-reperfusion injury by inhibiting activation of IKKbeta and inflammatory response. Am J Nephrol 30: 287–294. doi:10.1159/000225928.

9. Cochrane J, Williams BT, Banerjee A, Harken AH, Burke TJ, et al. (1999) Ischemic preconditioning attenuates functional, metabolic, and morphologic injury from ischemic acute renal failure in the rat. Ren Fail 21: 135–145.

10. Grenz A, Eckle T, Zhang H, Huang DY, Wehrmann M, et al. (2007) Use of a hanging-weight system for isolated renal artery occlusion during ischemic preconditioning in mice. Am J Physiol Renal Physiol 292: F475–F485. doi:10.1152/ajprenal.00275.2006.

11. Grenz A, Zhang H, Eckle T, Mittelbronn M, Wehrmann M, et al. (2007) Protective role of ecto-5’-nucleotidase (CD73) in renal ischemia. J Am Soc Nephrol 18: 833–845. doi:10.1681/ASN.2006101141.

12. Grenz A, Zhang H, Hermes M, Eckle T, Klingel K, et al. (2007) Contribution of E-NTPDase1 (CD39) to renal protection from ischemia-reperfusion injury. FASEB J 21: 2863–2873. doi:10.1096/fj.06-7947com.

13. Grenz A, Osswald H, Eckle T, Yang D, Zhang H, et al. (2008) The reno-vascular A2B adenosine receptor protects the kidney from ischemia. PLoS Med 5: e137. doi:10.1371/journal.pmed.0050137.

14. Guye M-L, Mc Gregor B, Weil G, Arnal F, Piriou V (n.d.) [Ischaemic and pharmacologic preconditioning: desflurane reduces renal reperfusion injury in rabbits]. Ann Fr Anesth Reanim 29: 518–523. doi:10.1016/j.annfar.2010.03.027.

15. Hernandez DJ, Roberts WB, Miles-Thomas J, Magheli A, Saha S, et al. (2008) Can ischemic preconditioning ameliorate renal ischemia-reperfusion injury in a single-kidney porcine model? J Endourol 22: 2531–2536. doi:10.1089/end.2008.0145.

16. Herrero F, Morales D, Baamonde C, Salas E, Berrazueta JR, et al. (2006) Ischemic preconditioning and kidney transplantation: in vivo nitric oxide monitoring in a rat ischemia-reperfusion experimental model. Transplant Proc 38: 2600–2602. doi:10.1016/j.transproceed.2006.08.044.

17. Hyodo Y, Miyake H, Kondo Y, Fujisawa M (2009) Downregulation of lectin-like oxidized low-density lipoprotein receptor-1 after ischemic preconditioning in ischemia-reperfused rat kidneys. Urology 73: 906–910. doi:10.1016/j.urology.2008.11.033.

18. Islam CF, Mathie RT, Dinneen MD, Kiely EA, Peters AM, et al. (1997) Ischaemia-reperfusion injury in the rat kidney: the effect of preconditioning. Br J Urol 79: 842–847.

19. Jang H-S, Kim J, Park Y-K, Park KM (2008) Infiltrated macrophages contribute to recovery after ischemic injury but not to ischemic preconditioning in kidneys. Transplantation 85: 447–455. doi:10.1097/TP.0b013e318160f0d1.

20. Jefayri MK, Grace PA, Mathie RT (2000) Attenuation of reperfusion injury by renal ischaemic preconditioning: the role of nitric oxide. BJU Int 85: 1007–1013.

21. Jiang SH, Liu CF, Zhang XL, Xu XH, Zou JZ, et al. (2007) Renal protection by delayed ischaemic preconditioning is associated with inhibition of the inflammatory response and NF-kappaB activation. Cell Biochem Funct 25: 335–343. doi:10.1002/cbf.1395.

22. Jiang S, Chen Y, Zou J, Xu X, Zhang X, et al. (2009) Diverse effects of ischemic pretreatments on the long-term renal damage induced by ischemia-reperfusion. Am J Nephrol 30: 440–449. doi:10.1159/000239574.

23. Joo JD, Kim M, D’Agati VD, Lee HT (2006) Ischemic preconditioning provides both acute and delayed protection against renal ischemia and reperfusion injury in mice. J Am Soc Nephrol 17: 3115–3123. doi:10.1681/ASN.2006050424.

24. Kadkhodaee M, Aryamanesh S, Faghihi M, Zahmatkesh M (2004) Protection of rat renal vitamin E levels by ischemic-preconditioning. BMC Nephrol 5: 6. doi:10.1186/1471-2369-5-6.

25. Kim J, Jang H-S, Park KM (2010) Reactive oxygen species generated by renal ischemia and reperfusion trigger protection against subsequent renal ischemia and reperfusion injury in mice. Am J Physiol Renal Physiol 298: F158–F166. doi:10.1152/ajprenal.00474.2009.

26. Kim J, Kim JI, Jang H-S, Park J-W, Park KM (2011) Protective role of cytosolic NADP(+)-dependent isocitrate dehydrogenase, IDH1, in ischemic pre-conditioned kidney in mice. Free Radic Res 45: 759–766. doi:10.3109/10715762.2011.577426.

27. Kinsey GR, Huang L, Vergis AL, Li L, Okusa MD (2010) Regulatory T cells contribute to the protective effect of ischemic preconditioning in the kidney. Kidney Int 77: 771–780. doi:10.1038/ki.2010.12.

28. Kosieradzki M, Ametani M, Southard JH, Mangino MJ (2003) Is ischemic preconditioning of the kidney clinically relevant? Surgery 133: 81–90. doi:10.1067/msy.2003.93.

29. Lazaris AM, Maheras AN, Vasdekis SN, Karkaletsis KG, Charalambopoulos A, et al. (2009) Protective effect of remote ischemic preconditioning in renal ischemia/reperfusion injury, in a model of thoracoabdominal aorta approach. J Surg Res 154: 267–273. doi:10.1016/j.jss.2008.06.037.

30. Lee HT, Emala CW (2000) Protective effects of renal ischemic preconditioning and adenosine pretreatment: role of A(1) and A(3) receptors. Am J Physiol Renal Physiol 278: F380–F387.

31. Lee HT, Emala CW (2001) Protein kinase C and G(i/o) proteins are involved in adenosine- and ischemic preconditioning-mediated renal protection. J Am Soc Nephrol 12: 233–240.

32. Li F-Z, Kimura S, Nishiyama A, Rahman M, Zhang G-X, et al. (2005) Ischemic preconditioning protects post-ischemic renal function in anesthetized dogs: role of adenosine and adenine nucleotides. Acta Pharmacol Sin 26: 851–859. doi:10.1111/j.1745-7254.2005.00109.x.

33. Liu L, Lin Y-qing, Yan L-tao, Hong K, Hou X-fei, et al. (2010) Extracellular ascorbic acid fluctuation during the protective process of ischemic preconditioning in rabbit renal ischemia-reperfusion model measured. Chinese Med J 123: 1441–1446.

34. Mahfoudh-Boussaid A, Badet L, Zaouali A, Saidane-Mosbahi D, Miled A, et al. (2007) [Effect of ischaemic preconditioning and vitamin C on functional recovery of ischaemic kidneys]. Prog Urol 17: 836–840.

35. Obal D, Dettwiler S, Favoccia C, Rascher K, Preckel B, et al. (2006) Effect of sevoflurane preconditioning on ischaemia/reperfusion injury in the rat kidney in vivo. Eur J Anaesthesiol 23: 319–326. doi:10.1017/S0265021505002000.

36. Ogawa T, Mimura Y, Kaminishi M (2002) Renal denervation abolishes the protective effects of ischaemic preconditioning on function and haemodynamics in ischaemia-reperfused rat kidneys. Acta Physiol Scand 174: 291–297.

37. Ogawa T, Nussler AK, Tuzuner E, Neuhaus P, Kaminishi M, et al. (2001) Contribution of nitric oxide to the protective effects of ischemic preconditioning in ischemia-reperfused rat kidneys. J Lab Clin Med 138: 50–58. doi:10.1067/mlc.2001.115648.

38. Orvieto MA, Zorn KC, Mendiola FP, Gong EM, Lucioni A, et al. (2007) Ischemia preconditioning does not confer resilience to warm ischemia in a solitary porcine kidney model. Urology 69: 984–987. doi:10.1016/j.urology.2007.01.100.

39. Park KM, Chen A, Bonventre JV (2001) Prevention of kidney ischemia/reperfusion-induced functional injury and JNK, p38, and MAPK kinase activation by remote ischemic pretreatment. J Biol Chem 276: 11870–11876. doi:10.1074/jbc.M007518200.

40. Park KM, Byun J-Y, Kramers C, Kim JI, Huang PL, et al. (2003) Inducible nitric-oxide synthase is an important contributor to prolonged protective effects of ischemic preconditioning in the mouse kidney. J Biol Chem 278: 27256–27266. doi:10.1074/jbc.M301778200.

41. Patschan D, Krupincza K, Patschan S, Zhang Z, Hamby C, et al. (2006) Dynamics of mobilization and homing of endothelial progenitor cells after acute renal ischemia: modulation by ischemic preconditioning. Am J Physiol Renal Physiol 291: F176–F185. doi:10.1152/ajprenal.00454.2005.

42. Salehipour M, Khezri A, Monabbati A, Jalaeian H, Kroup M, et al. (2007) Ischemic preconditioning protects the dog kidney from ischemia-reperfusion injury. Urol Int 79: 328–331. doi:10.1159/000109718.

43. Sola A, Palacios L, López-Martí J, Ivorra A, Noguera N, et al. (2003) Multiparametric monitoring of ischemia-reperfusion in rat kidney: effect of ischemic preconditioning. Transplantation 75: 744–749. doi:10.1097/01.TP.0000054683.72223.2D.

44. Song T, Peng Y-F, Guo S-Y, Liu Y-H, Liul L-Y (2007) Brief small intestinal ischemia lessens renal ischemia-reperfusion injury in rats. Comp Med 57: 200–205.

45. Sugino H, Shimada H, Tsuchimoto K (2001) Role of adenosine in renal protection induced by a brief episode of ischemic preconditioning in rats. Jpn J Pharmacol 87: 134–142.

46. Timsit MO, Gadet R, Ben Abdennebi H, Codas R, Petruzzo P, et al. (2008) Renal ischemic preconditioning improves recovery of kidney function and decreases alpha-smooth muscle actin expression in a rat model. J Urol 180: 388–391. doi:10.1016/j.juro.2008.02.043.

47. Toosy N, McMorris EL, Grace PA, Mathie RT (1999) Ischaemic preconditioning protects the rat kidney from reperfusion injury. BJU Int 84: 489–494.

48. Torras J, Herrero-Fresneda I, Lloberas N, Riera M, Ma Cruzado J, et al. (2002) Promising effects of ischemic preconditioning in renal transplantation. Kidney Int 61: 2218–2227. doi:10.1046/j.1523-1755.2002.00360.x.

49. Treska V, Molacek J, Kobr J, Racek J, Trefil L, et al. (2006) Ischemic training and immunosuppressive agents reduce the intensity of ischemic reperfusion injury after kidney transplantation. Exp Clin Transplant 4: 439–444.

50. Vianna PTG, Castiglia YMM, Braz JRC, Viero RM, Beier S, et al. (2009) Remifentanil, isoflurane, and preconditioning attenuate renal ischemia/reperfusion injury in rats. Transplant Proc 41: 4080–4082. doi:10.1016/j.transproceed.2009.09.078.

51. Wang Y-L, Zhao C-X, Jing Y-L, Zheng H-P, Cui G-J, et al. (2009) [The protective effects of ischemic preconditioning on the kidney injury following with ischemia/reperfusion of limbs and the possible mechanisms]. Zhongguo Ying Yong Sheng Li Xue Za Zhi 25: 492–495.

52. Wever KE, Warlé MC, Wagener FA, van der Hoorn JW, Masereeuw R, et al. (2011) Remote ischaemic preconditioning by brief hind limb ischaemia protects against renal ischaemia-reperfusion injury: the role of adenosine. Nephrol Dial Transplant: gfr103-. doi:10.1093/ndt/gfr103.

53. Wu M-S, Chien C-T, Ma M-C, Chen C-F (2009) Protection of ischemic preconditioning on renal neural function in rats with acute renal failure. Chin J Physiol 52: 365–375.

54. Xie J, Xie Z, Zhang S, Qi F (1999) [Protective effects of ischemic preconditioning on ischemia reperfusion injuries of kidney: experimental studies]. Hunan Yi Ke Da Xue Xue Bao 24: 316–318.

55. Yamashita J, Ogata M, Itoh M, Yamasowa H, Shimeda Y, et al. (2003) Role of nitric oxide in the renal protective effects of ischemic preconditioning. J Cardiovasc Pharmacol 42: 419–427.

56. Yamasowa H, Shimizu S, Inoue T, Takaoka M, Matsumura Y (2005) Endothelial nitric oxide contributes to the renal protective effects of ischemic preconditioning. J Pharmacol Exp Ther 312: 153–159. doi:10.1124/jpet.104.074427.

57. Yu Z, Wu Y, Guan H (1999) [Responses of normal rat kidney to different ischemic ways]. Zhonghua wai ke za zhi 37: 768–770.
