# Supplementary material for: Ischemic Preconditioning in the Animal Kidney, a Systematic Review and Meta-Analysis
Source: PLoS One. 2012 Feb 28;7(2):e32296. doi: 10.1371/journal.pone.0032296 (PMC3289650; doi:10.1371/journal.pone.0032296)
Supplement: Table S3 — Subgroup analysis serum creatinine. (DOC) [file pone.0032296.s003.doc]

| **Table S3 | Subgroup analysis serum creatinine** | | | | | | | |
| --- | --- | --- | --- | --- | --- | --- | --- |
| **Subgroup** | **n experiments** | **n studies** | **I^2^** | **n IRI only** | **n IRI + IPC** | **SMD and 95% confidence interval** |  |
| overall | 62 | 33 | 83% | 512 | 492 | 1.54 [1.16, 1.93] |  |
| early | 47 | 25 | 81% | 413 | 384 | 1.10 [0.72, 1.48] |  |
| late | 15 | 9 | 80% | 99 | 108 | 3.53 [2.45, 4.60] |  |
| continuous | 32 | 18 | 80% | 257 | 250 | 1.77 [1.25, 2.29] |  |
| fractionated | 30 | 20 | 85% | 255 | 242 | 1.31 [0.74, 1.87] |  |
| LIPC | 51 | 29 | 83% | 421 | 390 | 1.47 [1.03, 1.90] |  |
| RIPC | 6 | 3 | 79% | 60 | 60 | 1.53 [0.57, 2.48] |  |
| LIPC + RIPC | 5 | 1 | 76% | 31 | 42 | 2.48 [1.09, 3.87] |  |
| male | 42 | 22 | 79% | 345 | 339 | 1.51 [1.09, 1.93] |  |
| female | 2 | 2 | 24% | 17 | 18 | -0.03 [-0.83, 0.76] |  |
| male + female | 11 | 4 | 82% | 87 | 81 | 1.13 [0.25, 2.02] |  |
| mouse | 22 | 12 | 84% | 173 | 170 | 2.72 [1.88, 3.55] |  |
| rat | 35 | 18 | 78% | 303 | 286 | 1.02 [0.61, 1.44] |  |
| IRI = ischemia-reperfusion injury, IPC = ischemic preconditioning, SMD = standardized mean difference, LIPC = local ischemic preconditioning, RIPC = remote ischemic preconditioning | | | | | | | |
