# Supplementary material for: Ischemic Preconditioning in the Animal Kidney, a Systematic Review and Meta-Analysis
Source: PLoS One. 2012 Feb 28;7(2):e32296. doi: 10.1371/journal.pone.0032296 (PMC3289650; doi:10.1371/journal.pone.0032296)
Supplement: Table S4 — Subgroup analysis blood urea nitrogen. (DOC) [file pone.0032296.s004.doc]

| **Table S4 | Subgroup analysis blood urea nitrogen** | | | | | | | |
| --- | --- | --- | --- | --- | --- | --- | --- |
| **Subgroup** | **n experiments** | **n studies** | **I^2^** | **n IRI only** | **n IRI + IPC** | **SMD and 95% confidence interval** |  |
| overall | 29 | 17 | 76% | 242 | 241 | 1.42 [0.97, 1.87] |  |
| early | 22 | 12 | 75% | 197 | 185 | 1.20 [0.72, 1.68] |  |
| late | 7 | 5 | 75% | 45 | 56 | 2.45 [1.24, 3.66] |  |
| continuous | 11 | 8 | 79% | 94 | 103 | 2.04 [1.19, 2.89] |  |
| fractionated | 18 | 12 | 71% | 148 | 138 | 1.08 [0.57, 1.59] |  |
| LIPC | 20 | 14 | 81% | 167 | 155 | 1.50 [0.86, 2.14] |  |
| RIPC | 6 | 3 | 54% | 60 | 60 | 1.30 [0.69, 1.92] |  |
| LIPC + RIPC | 3 | 1 | 12% | 15 | 26 | 1.50 [0.69, 2.31] |  |
| male | 27 | 15 | 74% | 228 | 227 | 1.27 [0.83, 1.71] |  |
| mixed | 2 | 2 | 67% | 14 | 14 | 3.94 [1.38, 6.51] |  |
| mouse | 6 | 4 | 77% | 33 | 44 | 3.05 [1.45, 4.65] |  |
| rat | 21 | 12 | 60% | 186 | 174 | 0.93 [0.55, 1.30] |  |
| IRI = ischemia-reperfusion injury, IPC = ischemic preconditioning, SMD = standardized mean difference, LIPC = local ischemic preconditioning, RIPC = remote ischemic preconditioning | | | | | | | |
