# Supplementary material for: Ischemic Preconditioning in the Animal Kidney, a Systematic Review and Meta-Analysis
Source: PLoS One. 2012 Feb 28;7(2):e32296. doi: 10.1371/journal.pone.0032296 (PMC3289650; doi:10.1371/journal.pone.0032296)
Supplement: Table S5 — Subgroup analysis histology. (DOC) [file pone.0032296.s005.doc]

| **Table S5 | Subgroup analysis histology** | | | | | | | |
| --- | --- | --- | --- | --- | --- | --- | --- |
| **Subgroup** | **n experiments** | **n studies** | **I^2^** | **n IRI only** | **n IRI + IPC** | **SMD and 95% confidence interval** |  |
| overall | 26 | 15 | 63% | 205 | 191 | 1.12 [0.89, 1.35] |  |
| early | 23 | 13 | 55% | 180 | 168 | 1.01 [0.77, 1.25] |  |
| late | 3 | 3 | 76% | 25 | 23 | 2.50 [1.64, 3.35] |  |
| continuous | 12 | 6 | 73% | 104 | 95 | 1.20 [0.87, 1.54] |  |
| fractionated | 14 | 10 | 51% | 101 | 96 | 1.03 [0.71, 1.35] |  |
| LIPC | 25 | 14 | 58% | 193 | 179 | 1.04 [0.81, 1.28] |  |
| male | 20 | 10 | 70% | 166 | 156 | 1.04 [0.79, 1.30] |  |
| mixed | 4 | 4 | 0% | 25 | 23 | 1.46 [0.78, 2.14] |  |
| mouse | 7 | 6 | 5% | 49 | 44 | 1.66 [1.15, 2.17] |  |
| rat | 19 | 9 | 68% | 156 | 147 | 0.97 [0.71, 1.23] |  |
| IRI = ischemia-reperfusion injury, IPC = ischemic preconditioning, SMD = standardized mean difference, LIPC = local ischemic preconditioning, RIPC = remote ischemic preconditioning | | | | | | | |
