# Supplementary material for: Ischemic Preconditioning in the Animal Kidney, a Systematic Review and Meta-Analysis
Source: PLoS One. 2012 Feb 28;7(2):e32296. doi: 10.1371/journal.pone.0032296 (PMC3289650; doi:10.1371/journal.pone.0032296)
Supplement: Appendix S1 — Full search strategy for PubMed and EMBASE. (DOC) [file pone.0032296.s006.doc]

| **Appendix S1 | Full search strategy for PubMed and EMBASE** | | |
| --- | --- | --- |
| *PubMed* | Kidney | "kidney"[MeSH Terms] OR "acute kidney injury"[MeSH Terms] OR "kidney"[Tiab] OR "kidneys"[Tiab] OR "renal"[Tiab] OR "kidney transplantation"[MeSH Terms] OR "nephrology"[MeSH Terms] OR "nephrology"[Tiab] |
|  | Preconditioning | "ischemic preconditioning"[MeSH Terms] OR "IPC"[tiab] OR "RIPC"[tiab] OR "brief ischemia"[tiab] OR "brief ischaemia"[tiab] OR "preconditioning"[tiab] OR "pre conditioning"[tiab] OR "pre-conditioning"[tiab] OR "transient ischaemia"[tiab] OR "transient ischemia"[tiab] OR "intermittent ischaemia"[tiab]OR "intermittent ischemia"[tiab] OR "continuous ischemia"[tiab] OR "continuous ischaemia"[tiab] |
|  | Ischemia reperfusion injury | "warm ischemia"[Mesh Terms] OR "warm Ischemia"[Tiab] OR "warm Ischaemia"[Tiab] OR "cold ischemia"[Mesh Terms] OR "cold ischemia"[Tiab] OR"cold ischaemia"[Tiab] OR "primary graft dysfunction"[Mesh Terms] OR "primary graft dysfunction"[Tiab] OR "I/R"[Tiab] OR "IRI"[Tiab] OR "ischemic reperfusion"[Tiab] OR "ischaemic reperfusion"[Tiab] OR“ischemia reperfusion"[Tiab] OR“ischaemia reperfusion"[Tiab] OR “kidney ischemia"[Tiab] OR “kidney ischaemia"[Tiab] OR "renal ischaemia"[tiab] OR "renal ischemia"[tiab] OR "reperfusion injury"[Mesh Terms] OR "reperfusion injury"[tiab] OR "reperfusion injuries"[tiab] OR "ischemia reperfusion"[tiab] OR "ischaemia reperfusion"[tiab] OR "renal injury"[tiab] OR "renal injuries"[tiab] |
|  | Animals | Laboratory animal search filter [1] |
| *Embase* | Kidney | exp kidney/ OR exp acute kidney failure/ OR exp kidney transplantation/ OR exp kidney allograft rejection/ OR (renal OR kidney OR kidneys OR nephrology).ti,ab. |
|  | Preconditioning | exp ischemic preconditioning/ OR (IPC OR RIPC OR brief ischemia OR brief ischaemia OR preconditioning OR pre conditioning OR pre-conditioning OR transient ischaemia OR transient ischemia OR intermittent ischaemia OR intermittent ischemia OR continuous ischemia OR continuous ischaemia).ti,ab. |
|  | Ischemia reperfusion injury | exp reperfusion injury/ OR exp cold ischemia/ OR exp primary graft dysfunction/ OR (warm ischemia OR warm ischaemia OR cold ischemia OR cold ischaemia OR reperfusion injury OR primary graft dysfunction OR I/R OR IRI OR ischemic reperfusion OR ischaemic reperfusion OR kidney ischemia OR kidney ischaemia OR renal ischaemia OR renal ischemia OR reperfusion injury OR reperfusion injuries OR ischemia reperfusion OR ischaemia reperfusion OR renal injury OR renal injuries).ti,ab. OR (cold ischemia OR cold ischemia time OR cold ischemia times OR cold ischemic time OR cold ischemic times OR cold ischaemia OR cold ischaemia time OR cold ischaemia times OR cold ischaemic time OR cold ischaemic times).ti,ab. OR (warm ischemia OR warm ischaemia).ti,ab. |
|  | Animals | Laboratory animal search filter [2] |

**References**

1. Hooijmans CR, Tillema A, Leenaars M, Ritskes-Hoitinga M (2010) Enhancing search efficiency by means of a search filter for finding all studies on animal experimentation in PubMed. Lab Anim 44: 170–175. doi:10.1258/la.2010.009117.

2. de Vries RBM, Hooijmans CR, Tillema A, Leenaars M, Ritskes-Hoitinga M (2011) A search filter for increasing the retrieval of animal studies in Embase. Lab Anim 45: 268–270. doi:10.1258/la.2011.011056.
